# Exposure Assessment of Essential and Potentially Toxic Metals in Wheat-Based Sweets for Human Consumption: Multivariate Analysis and Risk Evaluation Studies

**DOI:** 10.3390/molecules28217365

**Published:** 2023-10-31

**Authors:** Mahmood Ahmed, Syed Salman Shafqat, Amna Javed, Mudassar Sanaullah, Abdul Shakoor, Muhammad Imtiaz Shafiq, Syeda Kiran Shahzadi, Tanveer A. Wani, Seema Zargar

**Affiliations:** 1 Department of Chemistry, Division of Science and Technology, University of Education, College Road, Lahore 54770, Pakistan; salman.shafqat@ue.edu.pk (S.S.S.); amnajaved0107@gmail.com (A.J.); mudassarsanaullah312@gmail.com (M.S.); 2 CSH Pharmaceuticals (Pvt.) Ltd., Ferozepur Road, Lahore 54000, Pakistan; chemistpbu@gmail.com; 3 School of Chemistry, University of the Punjab, Lahore 54590, Pakistan; imtiaz.ibb@pu.edu.pk; 4 Center for Bioinformatics and Drug Designing, University of the Punjab, Lahore 54590, Pakistan; 5 Department of Physiology, Faculty of Medicine and Health Sciences, McGill University, Montreal, QC H3A 0G4, Canada; kiran.syyed@gmail.com; 6 Department of Pharmaceutical Chemistry, College of Pharmacy, King Saud University, P.O. Box 2457, Riyadh 11451, Saudi Arabia; 7 Department of Biochemistry, College of Science, King Saud University, P.O. Box 222452, Riyadh 11451, Saudi Arabia; szargar@ksu.edu.sa

**Keywords:** food safety, metal toxicity, chemometric approach, daily intake, human health risk

## Abstract

In recent years, there has been a growing concern about the negative impact of unforeseen contaminants such as metals in commonly consumed food items, which pose a threat to human well-being. Therefore, it is of utmost importance to evaluate the levels of these contaminants to guarantee the safe consumption of these food items. The goal of the current research is to determine the levels of essential (EMs: Mg, Ca, Mn, Fe, Co, Cu, and Zn) and potentially toxic metals (PTMs: Al, Cr, Ni, As, Cd, and Pb) in various brands of wheat-based sweets. One hundred samples were collected and analysed via flame atomic absorption spectrometry (FAAS) and inductively coupled plasma–optical emission spectrometry (ICP-OES). Also, the current study was to investigate the distribution, correlation, and multivariate analysis of 13 metals (Mg, Ca, Mn, Fe, Co, Cu, Zn, Al, Cr, Ni, As, Cd, and Pb). Hierarchical cluster analysis (HCA) and principal component analysis (PCA) were used to interpret the metals’ association. The concentration (mg/kg) ranges of EMs were, in order, Mg (12.70–65.67), Ca (24.02–209.12), Mn (1.32–9.61), Fe (4.55–111.23), Co (0.32–8.94), Cu (2.12–8.61), and Zn (2.60–19.36), while the concentration (mg/kg) ranges of PTMs were, in order, Al (0.32–0.87), Cr (0.17–5.74), Ni (0.36–1.54), Cd (0.16–0.56), and Pb (0.14–0.92), and As was not detected in any sample under investigation. The HCA data revealed that Co, Al, and Ni form clusters with other metals. Sweets are prepared at high temperatures, and the elevated temperatures can increase the likelihood of Ni and Al leaching from stainless steel. Tolerable dietary intake (TDI) values for Ni were higher than the values established by the European Food Safety Authority (EFSA). The CR value found for the Ni and Cr was at the threshold level of cancer risk, if an amount of 25 g were to be used over a lifetime. In a nutshell, this study highlights the monitoring of EM and PTM levels in wheat-based sweets, and from a food safety perspective, the study is important for consumers of wheat-based sweets.

## 1. Introduction

Traditional sweets have been a favorite food among many cultures all over the world for a very long time. In the realm of culinary traditions, the sweet delicacies of the Indian subcontinent hold a distinct and revered place. These confections, collectively known as “mithai”, encompass a wide array of shapes, colors, and flavors, reflecting the rich cultural tapestry and diverse regional influences of India and Pakistan. During their preparation, various types of ingredients are used alongside wheat, such as cardamom, butter, oil, sugar, milk, and ghee (clarified butter). Different varieties of traditional sweets are available in Pakistan, each distinguished by its unique characteristics. These traditional sweets in Pakistan and India are not only an important part of festivals but also a part of day-to-day consumption throughout life. Despite their widespread consumption and demand, these sweets are extremely susceptible to contamination with various kinds of metals, including EMs and PTMs. These metals generally enter the sweets during their preparation process because the ingredients from which they are prepared might be contaminated with metals, which can lead to metal toxicity in humans after ingestion [1]. EMs at trace levels are required for proper human growth, development, and well-being and the proper functioning of living organisms [2,3]. PTMs are also found in different food items, but their safety levels are defined by health regulatory bodies such as the Food and Drug Administration (FDA) and the World Health Organization (WHO) [4,5]. Long-term exposure to these metals has been linked to numerous adverse health effects in humans, including mutagenic and teratogenic effects, bone diseases, cardiovascular disease, infertility, neurotoxicity, kidney problems, depression, hypertension, mental disorders, gastrointestinal cancer, gastric ulcers, psychological disorders, sideroblastic anemia, numerous liver problems, and loss of taste, smell, and appetite, among other problems [6]. Although the hazardous intake levels of EMs and PTMs have been documented by health regulatory bodies for various food items, to the best of our knowledge, there is no information available for wheat-based sweets.

Accurate evaluation of metals at trace level can be challenging because of their low concentration, which requires highly sensitive and precise analytical techniques to measure in order to obtain reliable results. Generally, atomic spectroscopic techniques such as atomic absorption spectrometry, in which flame (FAAS, flame atomic absorption spectroscopy) and graphite furnaces (ET-AAS, electrothermal atomic absorption spectroscopy) are used as ionization sources to determine the concentration of metals at ppm to ppb levels. By contrast, inductively coupled plasma spectroscopy with optical emission (ICP-OES) and with mass spectrometry (ICP-MS) are also used to estimate metal content at ultra-trace levels [7,8,9]. Given the widespread consumption of wheat-based sweets by a significant portion of the population over vast geographical areas, the current goal of this study is to look into the sweets’ content of Mg, Ca, Mn, Fe, Co, Cu, Zn, Al, Cr, Ni, As, Cd, and Pb. FAAS was employed for the determination of Mg, Ca, Mn, Fe, Cu, and Zn, whereas Al, Cr, Co, Ni, As, Cd, and Pb were determined via ICP-OES. This research aims to raise awareness about hygiene and evaluate health risks associated with consumption of wheat-based sweets. To characterize metals’ impact on human health, the estimated daily intake (EDI), target hazard quotient (THQ), hazard index (HI), cancer risk (CR), and cumulative cancer risk (CCR) were taken into account. 

## 2. Results and Discussions

### 2.1. Quality Control

Accuracy is a crucial and primary aspect of research because analytical methods are susceptible to certain errors that produce results deviating from the actual amounts of the elements. The ability to make judgments based on findings has an impact on the results acquired. NIST SRM 2384 was analysed as a part of recovery studies to ensure the precision of calculating the quantities of Mg, Ca, Mn, Fe, Co, Cu, Zn, Al, Cr, Ni, As, Cd, and Pb in actual samples. The outcomes are displayed as percentages of recovery in (Table 1). The remarkable extraction efficiency of the corresponding metals was demonstrated in the obtained recovery figures, which ranged from 98.2% to 101.4%.

The linear calibration curve of standards for each metal was plotted in the form of (y = mx + c) by plotting the absorbance against the concentration, where “m” indicated the calibration curve’s slope, “c” represented the intercept, and “x” represented the concentration of metal. The linear regression equation was established, and the essential parameters were tabulated. LOD and LOQ were determined via the proposed methods (Table S1). The methods demonstrated sufficient sensitivity as evidenced by the lower values of detection limits.

### 2.2. Application to Real Samples

Research on this topic is extremely limited, and to the best of our knowledge, no previous work has been carried out on this topic. The analysis of wheat-based sweet samples (SG, SB, SRW, SRP, SS, SP, SL, SDB, ST, and SPe) reveals the degree of variability of EMs and PTMs (Table 2).

#### 2.2.1. Magnesium

**Figure 1:**
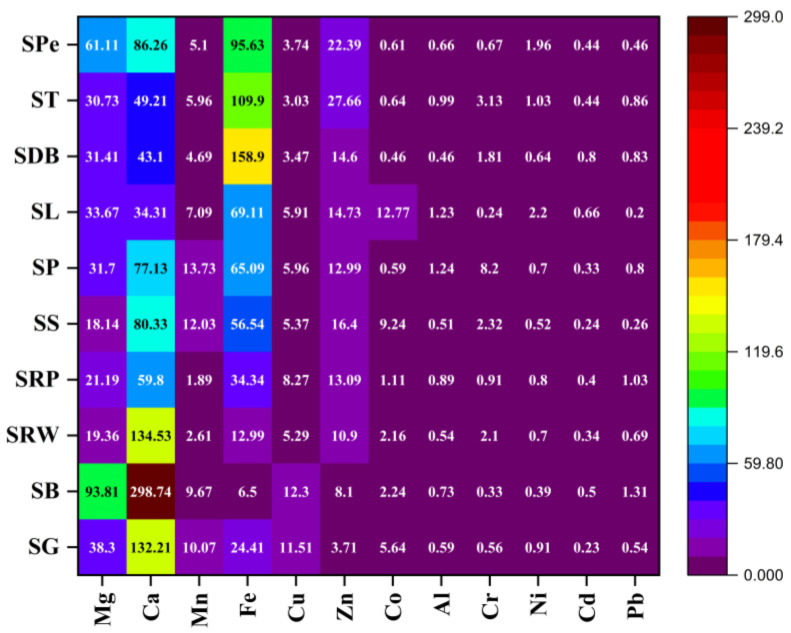
Demonstration of DI of EMs and PTMs in sweets (mg/day).

The mean values of Mg in different sweet samples ranged from 12.7 to 65.7 mg/kg (Table 2). The maximum value was observed in SB samples (85.25 mg/kg), while the minimum of 2.6 mg/kg was present in SRW. In our study, the DI values ranged between 18.14 and 93.81 mg (Figure 1), which is lower than the recommended daily allowance (RDA) of Mg at 420 mg/day. Mg also plays a role in the occurrence of arteriosclerosis, diabetes, obesity, metabolic syndrome, and hypertension. A diet rich in Mg can create an alkaline environment, reducing the excretion of Ca with consequent increase of bone density. Its deficiency is the cause of most depression and related mental health problems [10,11]. 

#### 2.2.2. Calcium

Ca plays a vital role in the prevention of cancer and maintaining the strength, health, and functionality of bones [12]. Ca, by abundance, is the fifth element in the earth’s crust. It has been identified in a variety of substrates and compounds, each of which has a variety of effects, the majority of which are beneficial to human health. Ca is essential for bone development; in fact, almost all of it in the human body is found in bones and teeth. Several studies have revealed that adequate intake of this metal keeps bones strong in the elderly and prevents and cures a variety of bone-related diseases such as osteoporosis [12]. The mean values of Ca in our sweet samples ranged from 24.0 to 209.1 mg/kg. The highest and lowest concentrations were found in SB and SG samples with the values 630.2 mg/kg and 10.6 mg/kg, respectively (Table 2). Ca has an RDA of 1000 mg/day; however, our study found DI values in the range of 34.31–298.74 mg (Figure 1), which is much lower than the RDA.

#### 2.2.3. Manganese

The mean Mn levels in various wheat-based sweet samples ranged from 1.3 to 9.6 mg/kg. SP and SRP samples had the highest and lowest concentrations, with values of 14.5 mg/kg and 0.11 mg/kg, respectively (Table 2). Mn has an RDA of 2.3 mg/day; however, our study found DI values in the range of 1.89–13.73 mg (Figure 1). Mn is present in various enzymes, including peptidases, phosphatases, arginase, phosphoglucomutase, and glucosyl transferases [11,13]. Excessive amounts of Mn lead to impaired cognitive development and result in neurotoxicity and even Parkinson’s disease [11,13]. Cardamom and nuts are popular spices used for flavoring, which could be the origin of toxic metals in wheat-based sweets. Cardamom itself is unlikely to be a major contributor to metal levels in sweets, but the soil in which these plants are grown can influence their metal content. The agricultural sector applies numerous chemical fertilizers and pesticides which may be a major factor in soil becoming contaminated with metals.

#### 2.2.4. Iron

The mean values of Fe ranged from 4.6 to 111.2 mg/kg in wheat-based sweet samples. The minimum result was found in SB samples (0.12 mg/kg) while the maximum was found in ST samples (336.55 mg/kg) (Table 2). The RDA of Fe is given as 8 mg/day, and the present study found DI values in the range of 6.50–158.90 mg (Figure 1). Fe contamination can occur during food processing and handling. For instance, Fe-containing utensils, tools, or storage containers used in the preparation of food may leach Fe into the finished product. To add Fe to food products, certain compounds with Fe can be utilized as food additives. While this is done to treat Fe deficiency in a population, using too much of these additives or formulating them incorrectly might cause Fe contamination [14]. Excess Fe can be dangerous because it hastens the formation of oxygen radicals via the Fenton or Haber–Weiss reactions [15,16]. 

#### 2.2.5. Copper

The mean value of Cu in wheat-based sweets was found in the range of 2.1 to 8.6 mg/kg. The highest and lowest concentrations were found in SB (12.0 mg/kg) and SP (0.25 mg/kg) samples, respectively (Table 3). Cu has an RDA of 0.9 mg/day; however, in our study, DI values were found in the range of 3.03–12.30 mg (Figure 1), which is much more than the recommended intake. The observed mean descending concentration pattern in different samples was SB > SG > SRP > SP > SL > SS > SRW > SPe > SDB > ST (Table 2). Although it is an essential micronutrient for humans, high levels can be toxic and trigger redox Fenton-type reactions, which damage and ultimately destroy oxidative cells. Acute Cu poisoning in humans can cause gastrointestinal problems such as nausea or abdominal pain [9,17,18]. Cu can enter the food chain via tainted food, processed food, and soil that has been mineralized by agriculture. Cu can be found in trace amounts in soil, water, and plant materials, and if cows consume feed or water that is naturally higher in Cu, it may lead to slightly higher Cu levels in their milk. Nuts, which are another common ingredient in wheat-based sweets, can also have high levels of Cu.

#### 2.2.6. Zinc

In wheat-based sweet samples, the mean Zn levels ranged from 2.6 to 19.0 mg/kg. The highest and lowest concentrations were found in ST (67.7 mg/kg) and SG (1.8 mg/kg) samples, respectively (Table 2). Zn has an RDA of 11 mg/day, but in our study, the DI values were found in the range of 3.71–27.66 mg (Figure 1). Zn participates in regulation of gene expression and in immune system function. It is involved in diverse biological functions, such as homeostasis, immune response, and oxidative stress [19]. Imbalance in trace amounts of Zn leads to loss of appetite, inhibition of growth, skin changes, somatic effects, and immunological abnormalities [3,9,17,18,20]. Zn levels in crops may arise as a result of the application of certain fertilizers or soil amendments that contain Zn. The quality of water used in the preparation of sweets is crucial. Depending on its source, water can carry trace amounts of metals, including Zn. Water quality, as well as the type of pipes and containers used for storage, could be the source of Zn [21]. 

#### 2.2.7. Cobalt

The mean values of Co were obtained in the range of 0.32–9.0 mg/kg in wheat-based sweet samples. The highest and lowest concentrations were found in SL and SRP samples with the values 14.3 mg/kg and 0.11 mg/kg, respectively (Table 2). In our study, DI values (Figure 1) were found in the range of 0.46–12.77 mg when sweets were consumed at a rate of 100 g of sweet/day, which is much higher than the oral threshold given by the European Food Safety Authority (EFSA). Co revealed the mean concentration pattern as SL > SS > SG > SB > SRW > SRP > ST > SPE > SP > SDB. According to the EFSA [22], a health-based recommendation for chronic exposure to Co would be 1.6 μg/kg bw/day for oral Co threshold effects. Co can enter the environment as a result of specific industrial operations. Examples include the discharge of Co particles into the air and water during metal smelting, manufacturing, and mining operations. These particles can eventually contaminate crops and make their way into food. Co can leach into food when materials containing Co are utilized in food processing or packaging. Co serves as a metallic element in vitamin B12, which is essential for DNA control, protein synthesis, and brain and nervous system health. However, after prolonged exposure, Co is considered harmful to the human body [23]. High Co levels may have a negative effect on respiratory health and also result in the formation of inflammatory fluid or osteolysis. Co levels in the blood or tissue determine if these systems are involved in dangerous reactions. Both peripheral and cerebral impairments may result from Co-related neurotoxicity [24]. 

#### 2.2.8. Aluminum

In sweet samples, the mean values of Al were found to range from 0.32 to 0.87 mg/kg. The highest and lowest concentrations (Table 2) were found in SL and SDB samples, having the values of 1.1 mg/kg and 0.11 mg/kg, respectively. The WHO considers the PTWI of Al to be 2 mg/kg bw, whereas the EFSA has given a TWI of 1 mg/kg bw for Al from all sources, including food additives [25,26]. The present study found a DI value of Al in the range of 0.46–0.99 mg from 100 g of sweets (Figure 1). Al is not nutritionally important, and a high concentration may lead to various type of ailments in humans [27,28]. The toxic effects of Al are primarily due to its pro-oxidant activity, which causes oxidative stress, and oxidation of cellular proteins and lipids, ultimately leading to denaturation or conformational or structural alterations. Exposure to Al has been associated with the development of Alzheimer’s disease [27,28,29]. The brain, bones, and kidney are just a few of the many tissues where Al can build up and cause damage and dysfunction. The stainless steel equipment and utensils are often used in food preparation such as the cooking of sweets, and thus Al may leach from cookware. Al foils are frequently used to cover sweets in local markets, which can possibly be a source of Al [30].

#### 2.2.9. Chromium

The mean values in the samples under investigation were obtained in the range of 0.17–5.7 mg/kg. SPe samples had the minimum value (0.11 mg/kg), while on the other hand, SP showed highest concentration (7.5 mg/kg) (Table 2). The DI values were obtained in the range of 0.33 to 8.20 mg (Figure 1). The EFSA established a TDI of 0.30 mg/kg bw for Cr(III) due to its effects on reproduction. The tannery industry may have contributed to Cr contamination in foodstuffs [31]. The toxicological effects of Cr can be classified into multiple groups, including respiratory, carcinogenic, genotoxic, mutagenic, cardiovascular, reproductive, and developmental [32]. Cr(VI) is more carcinogenic than Cr(III), causing redox homeostasis disruption, and damages DNA–protein cross-linkage. Cr(III) is employed in numerous dietary supplements as a vital micronutrient, typically at low concentrations. An excess of Cr(III) and the presence of Cr(VI) all contribute to the development of dermatitis, eczematous skin reactions, nasal ulcers, squamous cell carcinoma, gastro-enteritis, and inhibition of collagen secretion, which promotes bone fracture healing [33,34]. Nuts, e.g., almonds and walnuts, and whole grains, which are used as ingredients in sweets, may contain trace amounts of Cr and ultimately contaminate the final products. Some leavening agents, such as baking powder or baking soda, may contain trace amounts of Cr, and they can contribute to contaminate the sweets [35].

#### 2.2.10. Nickel

In the current study, the mean concentration values of Ni were found in a range from 0.27 to 1.5 mg/kg. SP samples showed lowest values of 0.11 mg/kg, and SPe samples were found to have the maximum value at 1.6 mg/kg (Table 2). The observed pattern of mean concentration was SL > SPe > ST > SG > SRP > SRW = SP > SDB > SS > SB. In the present study, DI values were found in range of 0.39-1.96 mg as presented Figure 1. The EFSA considers 0.013 mg/kg bw the safe TDI of Ni [36], so our results show a higher value of DI than that established as safe by the EFSA. Ni is a typical trace element that is present in the biosphere, water, air, and soil. Ni is also found in living organisms, particularly in plants. Ni absorption via the oral route results in acute toxicity symptoms in the human body [37,38]. High concentrations may causes immunotoxicity and skin allergies and damages and interfere with the base excision repair property of DNA [37,38]. Cross-contamination can occur if utensils, equipment, or surfaces that have previously come into contact with Ni-containing foods are used in preparation. Some ingredients, such as nuts (e.g., almonds, pistachios), may naturally contain trace amounts of Ni. The soil in which these nuts are grown and the water used for irrigation can influence their metal content [39].

#### 2.2.11. Cadmium

In the current study, the mean concentration values of Cd were found in a range from 0.16 to 0.56 mg/kg in different sweet samples. The minimum value of 0.11 mg/kg was found in SG, SRP, and SS samples while the maximum value of 0.16 mg/kg was shown by SPe (Table 2). DI values were found in the range of 0.23–0.66 mg (Figure 1). The mean concentration pattern was SDB > SL > SB > ST = SPe > SRP > SRW > SP > SS > SG. FAO/WHO has established a 25 μg/kg bw Cd as PTMI [26], whereas the TWI of Cd is given by the EFSA as 2.5 μg/kg bw [40]; consumers must be cautious about the excess long-term consumption of the sweets under study. Exposure to Cd has both acute and chronic effects, including kidney and skeletal harm and may cause cancer in humans [41,42]. Cd is an extremely hazardous metal, and its high concentrations cause health issues such as high blood pressure, nerve damage, behavioral disorders, dementia, and reduced learning ability [43,44]. In some sweet recipes, palm products such as palm oil or palm sugar may be used. The metal content in these products can vary based on the source and processing methods. For example, palm oil may contain trace amounts of Cd [45]. 

#### 2.2.12. Lead

The mean concentrations of Pb were found in the range of 0.14–0.9 mg/kg. The minimum value of 0.14 mg/kg was found in Spe, while 2.1 mg/kg was the highest value exhibited by the sample SB (Table 2). DI values were found in the range of 0.20–1.31 mg (Figure 1). The observed mean concentration pattern was SB > SRP > ST > SDB > SP > SRW > SG > Spe > SS > SL (Table 2). The EFSA has established a 15 μg/kg bw day BMDL_10_ for Pb. Pb is an extremely hazardous metal, and its high concentrations cause health issues such as high blood pressure, nerve damage, behavioral disorders, dementia, and reduced learning ability [43,44]. Because of its capacity to inhibit or mimic the action of Ca, Pb can cause changes in blood Ca level and influence protein kinase C, which regulates nerve excitability and storage of memory, even at picomolar concentrations [7]. Pollution from industrial activities or traffic emissions, for instance, can lead to the presence of metals such as Pb and Cd in food products. Sometimes, shopkeepers in local markets and local venders do not cover the sweets, which could be the source of contamination. Artificial or natural colorants and flavorings may contain trace amounts of Pb. While these are typically used in small quantities, they can contribute to the overall metal content if not carefully sourced [46].

### 2.3. Multivariate Analysis

**Figure 2:**
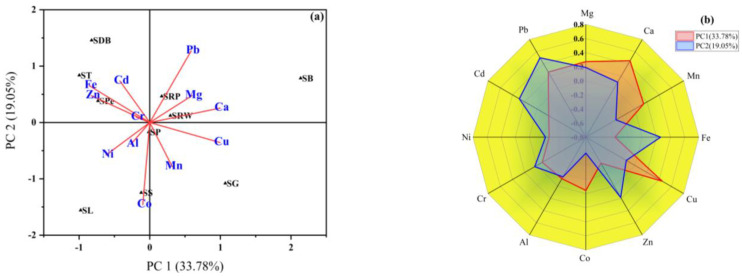
Principal component analysis (**a**) and radar diagram of loading values (**b**) of EMs and PTMs in wheat-based sweets.

The metals, including Mg, Ca, Mn, Cu and Pb, illustrated significant positive loading values ≥ 0.14 in PC1, accounting for 33.78% of the total variance. Besides that, in PC1, samples SG, SB, SRW, and SRP had the greater score values. Conversely, PC2 explained 19.05% of the total variation in terms of metals such as Mg, Ca, Fe, Zn, Cr, Cd, and Pb with positive loading values greater than 0.03. The samples SDB, ST, SPe, SRP, and SB were found to have greater score values in PC2 (Figure 2a). The PCA suggests that the metals Ca, Mn, Cu, and Pb in PC1 showed the higher loadings, while on the other hand, in PC2, Ca, Fe, Zn, Cr, Cd, and Pb had higher loadings, as shown in Figure 2b and Table S2, Supplementary Materials. Figure 2b summarizes the loading of Ca, Fe, Zn, Cr, Cd, and Pb among the Ems and PTMs under investigation in PC2, which make up 19.05% of the total variance.

**Figure 3:**
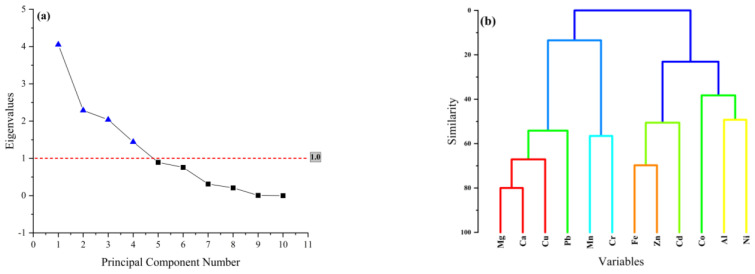
Scree plot (**a**) and hierarchical component analysis (**b**) of EMs and PTMs in wheat-based sweets.

As determined via principal component analysis, four principal components with eigenvalues greater than one represented the PCA of measured parameters by approximately 81.83% variance (Table S3, Supplementary Materials). Consequently, the component scree plots demonstrated that EMs and PTMs in sweets originating from four independent sources (Figure 3a). Therefore, HCA was used to conduct additional PCA verification, which is a technique used to classify a collection of metals into different correlated classes in a variable space. Figure 3b shows how the PCA results were associated in the HCA dendrogram, which revealed four distinct clusters or correlated groups. The first cluster contained Mg, Ca, and Cu, while the second cluster included Mn and Cr.

**Figure 4:**
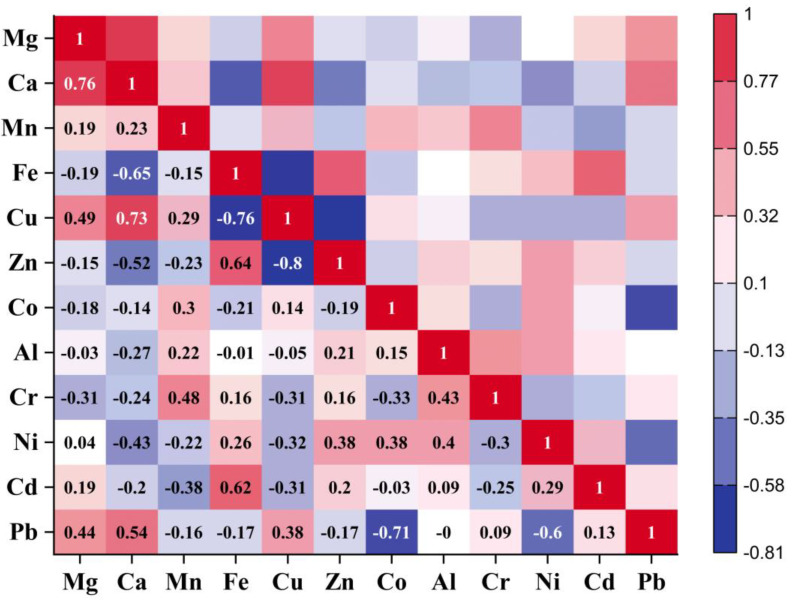
PCC demonstration of EMs and PTMs in wheat-based sweets.

Pearson’s correlation analysis or Pearson correlation coefficient (PCC) was used to determine the sequential combinations of the original of variables and the results are displayed in matrix form. PCC is effective for assessing the strength and direction of a link between variables. In other words, large levels of confidence correlation between metals (EMs and PTMs) indicate that these metals are derived from the same source. In other words, large levels of confidence or positive correlation between metals indicate that these metals are derived from the same source and have same origin. Therefore, PCC was entrusted with investigating whether the varied metal contents in different samples of sweets were related. Figure 4 illustrates the results of the PCC test, which highlights the significant connections between the following metals: Ca–Mg (r = 0.76), Cu–Mg (r = 0.49), Cu–Ca (r = 0.73), Zn–Fe (r = 0.64), Cr–Mn (r = 0.48), Cr–Al (r = 0.43), Ni–Zn (r = 0.38), Ni–Co (r = 0.38), Ni–Al (r = 0.4), Cd–Fe (r = 0.62), Pb–Mg (r = 0.44), Pb–Ca (r = 0.54) and Pb–Cu (r = 0.38). Significant correlations were found between metals within the same clusters and principal components, indicating that the results of PCA, HCA, and PCC were all supportive.

The PCA, HCA (Figure 2a and Figure 3b), and positive correlation value of 0.76 (Figure 4) between Ca and Mg clearly show that these metals are derived from the same source. The positive correlation of Cu with Mg and Ca demonstrates that milk, which is used in almost all kind of sweet recipes, could be the source of these three metals. The Cu content in milk is primarily influenced by factors such as the animal’s feed and the environment, such as soil composition and farming practices [47]. Significant positive correlation value of 0.48 (Figure 3) between Mn and Cr demonstrate that these metals originated from a reputable source. Some sweet recipes may use cooking oils or fats other than ghee or clarified butter. The quality and source of these oils can impact the metal content. For example, oil extracted from seeds or nuts may contain trace amounts of metals such as Mn and Cr. The third cluster showed that Fe, Zn, and Cd contamination could originate from a common source. The containers or equipment used for storing or processing butter can sometimes leach metals into the product. Ghee or clarified butter can introduce these metals if the dairy source and processing methods are not closely monitored [48]. Additionally, fertilizers and water can be the source of these metals. Some food colorants, flavorings, and preservatives may contain trace amounts of metals as impurities. These additives, if not properly sourced or regulated, can contribute to the metal content of sweets. Last but not least, Co, Al and Ni were the elements that comprised the fourth cluster. Sweets are prepared at high temperatures; this may lead to greater metal migration from cookware and utensils. Elevated temperatures can increase the likelihood of Ni and Al leaching from stainless steel. Additionally, Ni is used as a catalyst in the hydrogenation of oil into ghee, and trace amounts of Ni in ghee can contaminate wheat-based sweets because these sweets are cooked in ghee [49]. 

### 2.4. Calculation of Impact on Human Health

#### 2.4.1. Estimated Daily Intake

Table 3 presents the EDI of each metal from consuming wheat-based sweets as a first attempt to evaluate the risk to human health over a lifetime; the amount that was taken into account was set at 25 mg/day. The daily intake of essential and potentially toxic metals was estimated. The highest EDI value of 7.47 × 10^−2^ for Ca (SB) and lowest value of 9.36 × 10^−4^ for Cu (SPe) was obtained for EMs, while for PTMs the highest (2.05 × 10^−3^) and lowest (9.64 × 10^−5^) values of EDI were obtained for Cr(SP) and Ni (SB), respectively (Table 3). Frequently ingesting toxic metals at risky doses via food, such as wheat-based sweets, may have an impact on a number of biological and metabolic processes in humans. It was therefore believed that combining the anticipated degree of food contamination with the associated rate of food consumption could be a valuable tool for weighing the advantages and disadvantages. 

#### 2.4.2. Target Hazard Quotient

**Figure 5:**
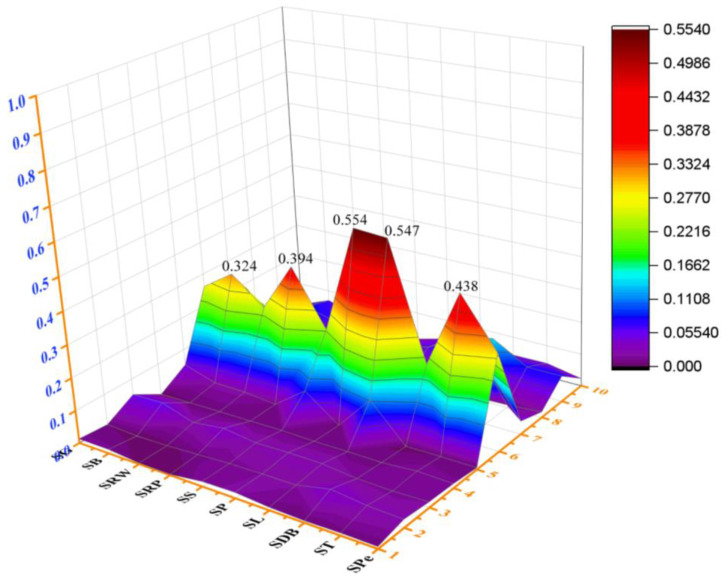
THQ representation of EMs and PTMs in wheat-based sweets.

All of the samples of what-based sweets had a THQ of less than 1. The highest values at 5.54 × 10^−1^ and 5.47 × 10^−1^ were observed for Al in SP and SL samples, respectively (Figure 5 and Table S4, Supplementary Materials). Figure 5 demonstrates that THQ values ware below hazard levels.

**Figure 6:**
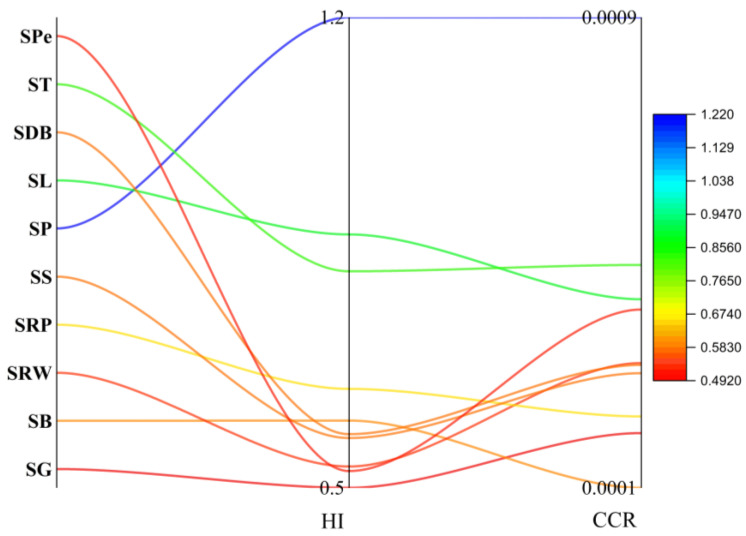
Results of HI and CCR of EMs and PTMs in wheat-based sweets.

The mean cumulative health risks were determined and categorized as HI by adding together the health risks of the nine tested metals. For the ingestion of sweets, the values of HI were found in a range between 0.5 and 1.2 (Figure 6). The HI values were higher than the threshold value 1 in SP samples. The findings showed that customers would be exposed to major potential health risks over the course of their entire lives if they consumed “Patisa” sweets. The higher concentration of metals was found in “Patisa”, which surpassed the threshold values of THQ and HI. In the production process of “Patisa”, the number of ingredients is greater than the other sweets under investigation. There is more chance of metal contamination in the final product. 

#### 2.4.3. Carcinogenic Risk and Cumulative Cancer Risk

**Figure 7:**
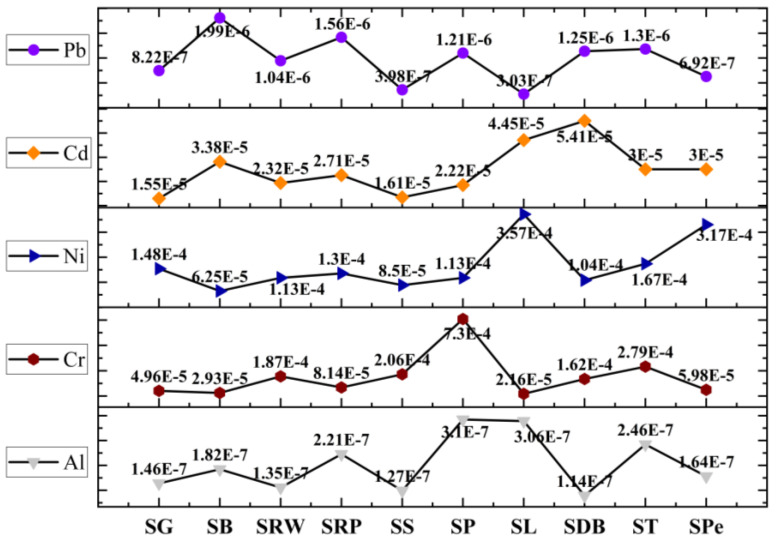
CR results variations of EMs and PTMs in wheat-based sweets.

CR content was evaluated by employing the carcinogenic slope factor (CSF) from a lifetime exposure to Al, Cr, Ni, Cd, and Pb via consumption of wheat-based sweets. The threshold value is 10^−4^, whereas the value ≥ 10^−3^ can be considered serious. In SRW, SP, SDB, and ST samples, Cr was found at threshold level (Figure 7), whereas the CR value for Ni was found to be at the threshold level of cancer risk for the all samples except SB and SS samples. The higher level of Ni might be due to ghee, which is an essential ingredient for the cooking of sweets. Therefore, the findings suggest that there was little chance of carcinogenic risk due to exposure to Ni from ingesting the wheat-based sweet samples under investigation, whereas the CCR values for all samples except SDB were found at threshold levels in the current investigation, which is alarming (Figure 7). For the CCR value, the major contributors are Cr and Ni, while the rest of the metals have a negligible role.

## 3. Materials and Methods

### 3.1. Reagents and Solutions

HNO_3_ (65% *m*/*m*) and H_2_O_2_ (30% *v*/*v*) were purchased from a local vendor (Pak Scientific Store, Lahore, Pakistan). Certified reference material (CRM) of each metal (1000 mg/L, Merck, Germany) was employed for instrument calibration after diluting it with HNO_3_ (2% *v*/*v*). Distilled water (18 MΩ·cm resistivity) for the dilution of all solutions was prepared via GenPure water system (Thermo Scientific, Waltham, MA, USA). All glassware and materials used in the procedure were soaked in HNO_3_ (20% *v*/*v*) and then exhaustively washed with distilled water. As a reference material, NIST SRM 2384 (baked chocolate) was used.

### 3.2. Sample Collection and Preparation

One hundred samples of traditional sweets from ten different brands were collected from different bakeries in Lahore, Pakistan. Ten sweet samples of each brand were collected (10 × 10 = 100) and transported to the lab. The sweets’ samples were labelled locally with characteristics as follows: gulab jamun (an iconic spherical sweet; ingredients: milk solids, flour, sugar, ghee, and saffron, coded as SG1-10), barfi (a firm, square or diamond-shaped sweet; ingredients: milk solids, flour, sugar, and cardamom, coded as SB1-10), white and pink rasgulla (a spongy, ball-shaped sweet; ingredients: milk solids, flour, sugar, pink color, and ghee, coded as SRW1-10 and SRP1-10 respectively), sohan halwa (brittle, square-shaped sweet; ingredients: milk solids, flour, sugar, corn flour, ghee, almonds, pistachios, and cardamom seeds, coded as SS1-10), Patisa (exhibits a flaky, golden appearance, ingredients: milk solids, flour, sugar, ghee, almonds, pistachios, cardamom seeds, and nut oils, coded as SP1-10), laddu (a ubiquitous spherical sweet with a golden-brown surface; ingredients: flour, sugar, ghee, almonds, pistachios, and cardamom seeds, yellow color, coded as SL1-10), tosha (honeycomb-like structure; ingredients: flour, sugar, ghee, cheese, and red color, coded as ST1-10), peda (a round sweet, exhibits a pale beige color; ingredients: flour, sugar, ghee, almonds, pistachios, cardamom seeds, and yellow color, coded as SPe1-10), and doda barfi (a firm, granular texture and a rich brown coloration; ingredients: flour, sugar, ghee, milk solids, and liquid glucose, coded as SDB1-10). The collected samples were homogenized in the laboratory by crushing them in a mortar with the help of pestle. Extraction of EMs and PTMs was carried out via acid digestion. A 0.5 g quantity of each sweet sample was taken for this purpose and was placed in 25 mL crucible. Then, 10 mL of 65% HNO_3_ was added to each sample. For 6–8 h, the mixture was heated in a heating mantle at 85 °C until the samples were completely digested and a solid residue was left behind. After that, 1 mL of 30% H_2_O_2_ was added to each sample, and samples were again heated in a heating mantle for 1 h at 85 °C. Then, an appropriate quantity of distilled water was added to the obtained filtrate for the purpose of making the final volume up to 25 mL. The SRM 2384 was also digested in the same manner, whereas stock solutions of real samples and SRM 2384 were further diluted as per requirement of analysis. The presence of EMs and PTMs in the sweet samples were determined by FAAS and ICP-OES. All the measurements were made in triplicate and presented in mg/kg. 

### 3.3. Instrumentation

FAAS, Model: PG-990 equipped with auto-sampler from PG-instruments, UK, was utilized for the analysis of Mg, Ca, Mn, Fe, Cu, and Zn after operational condition optimization. FAAS operational parameters used in the analysis were as follows: Wavelength (nm), Mn: 279.5, Fe: 248.3, Cu: 324.7, Zn: 213.9, Mg: 285.5, Ca: 422.7; slit width, 0.4 nm; lamp current, 5.0 mA; acetylene gas flow, 1700 mL/min. ICP-OES (iCAP 7400, Thermo Fisher Scientific, City, UK) with the radial viewed plasma equipped with echelle type 52.91 grooves/mm ruled grating was used for the determination of metals in wheat-based sweet samples. The operating conditions were as follows: RF power, 1250 W; radial viewing height, 8 mm; plasma coolant gas flow rate, 15.0 L/min; auxiliary gas flow rate, 1.0 L/min; nebulizer gas flow, 0.65 L/min, nebulizer type, V-groove, pressure 240 kPa; sample uptake rate, 1.5 mL/min; pump rate 35 rpm during flushing, 20 rpm during the analysis and spectral lines (nm) were selected as Al: 396.152, Cr: 267.716, Ni: 231.604, Co: 238.892, Cd: 214.439, As: 193.759, and Pb: 220.353 [34].

### 3.4. Quality Control

The accuracy and precision of the suggested approach for metal analysis in samples of coconut milk were assessed using NIST, SRM 2384 (baked chocolate). Studies on percentage recovery and relative standard deviation (% RSD) were carried out in replicates of ten (*n* = 10) in order to evaluate accuracy and precision. A blank measurement was taken in replicates of eighteen (*n* = 18) to determine the detection limits (LOD = 3 σ/S, LOQ = 10 σ/S) where σ and S are SD (standard deviation) of blank and slope of calibration curve respectively [5,48,50]. The calibration curve (y = mx + b) was built over a linear dynamic range of 2.0–10.0 μg/mL for Mg, Ca, Mn, Cr, Cu, Co, Ni, and Zn, 0.01–500 μg/L for Al, As, Cr, and Cd while 0.02–1000 μg/L for Pb (Table S1, Supplementary Materials). 

### 3.5. Statistical Analysis

Hierarchical cluster analysis (HCA) and principal component analysis (PCA), as well as a bivariate statistical test, were used to assess the data from this study. The Pearson correlation coefficient (PCC) was also used. HCA and PCC were used to interpret the metal grouping. The results of PCC and HCA were then utilized to minimize the size of the data sets and identify new features. IBM SPSS^®^ version 26.0 and OriginPro 2022 were the tools employed for all statistical work.

### 3.6. Calculation of Impact on Human Health

Several investigations have been conducted to investigate the route of potentially hazardous components to humans via consumption of contaminated food and beverages. In this study, the estimated daily intake (EDI), total hazard quotient (THQ), hazard index (HI), carcinogenic risk (CR), and cumulative cancer risk (CCR) were all used to calculate the impact of toxic metals found in coconut milk samples on human health.

#### 3.6.1. Estimated Daily Intake (EDI)

The maximum quantity of a chemical to which an individual may be exposed may be ingested every day over a lifetime without experiencing adverse consequences, making EDI a completely commonplace concept in chemical threat assessment. If consumed in excess, this amount may also result in poisonous effects. It is a quantitative estimate of the average oral exposure to the human population, and it is expressed in mg/kg/day [51]. Using the following formula, the EDI of the metals Ca, Mg, Fe, Cr, Mn, Co, Ni, Cu, Al, Zn, Cd, and Pb in mg/kg/day was calculated [16,52,53].

$$\mathrm{EDI}=\frac{\mathrm{Ingestion}\;\mathrm{rate}\;\left(g/\mathrm{day}\right)\times \mathrm{Metal}\;\mathrm{concentration}\;\left(\mathrm{mg}/\mathrm{kg}\right)}{\mathrm{BW}\;\left(\mathrm{kg}\right)}\times 10^{-3}$$

where the ingestion rate was considered 25 g/day over a lifetime period and 70 kg is, in the Pakistani population, an average adult weight. The EDI was calculated to access the lifetime hazard for the consumption of the sweet. The benchmark dose lower limit (BMDL) for Pb, the provisional tolerable monthly intake (PTMI) for Cd, the tolerable daily intake (TDI) for Cr, and Ni, and the health-based guidance for Co were all used in the calculation of daily intake (DI) in order to compare it to the values established for the available health-based guidance [40,54,55]. The DI was calculated based on the daily consumption of 100 g of sweet.

$$\mathrm{DI}=\frac{\mathrm{Consumption}\;\left(g/\mathrm{day}\right)\times \mathrm{Metal}\;\mathrm{concentration}\;\left(\mathrm{mg}/\mathrm{kg}\right)}{\mathrm{BW}\;\left(\mathrm{kg}\right)}$$

#### 3.6.2. Target Hazard Quotient (THQ) and Hazard Index (HI)

THQ is the ratio of a substance’s potential exposure to the extent where no adverse effects are expected, calculated by dividing the exposure by the appropriate persistent or acute value. In some studies, the THQ method for hazard characterization was used, and the estimation of risk was linked to the chemical substance concentrations in a specific food object [56]. The following formula was used to calculate THQ, which is typically used to estimate potential non-carcinogenic hazards.

$$\mathrm{THQ}=\frac{\mathrm{EF}\times \mathrm{ED}\times \mathrm{IR}\times \mathrm{MC}}{\mathrm{RfD}\times \mathrm{BW}\times {\mathrm{AT}}_{\mathrm{noncancer}}}\times 10^{-3}$$

where EF is exposure frequency (260 days/year), (ED) is the exposure duration (30 years for non-cancer risk as used by US EPA [57], RfD is the oral reference dose, BW stands for the average body weight (kg) for an adult (70 kg), and AT is average time for non-carcinogens (365 days/year × 30 years). The reference dose, RfD (mg/kg BW/day) values are as follows; Al = 0.0004, Cr = 0.003, Mn = 0.14, Fe = 0.7, Co = 0.02, Ni = 0.002, Cu = 0.04, Zn = 0.30, Cd = 0.001, As = 0.0003, and Pb = 0.0035 [58]. HI was calculated by summing up the THQ of each element under investigation was determined by the formula below. If HI is less than 1, no significant health risks are taken into account.

$$\mathrm{HI}=\sum_{i=1}^{n}{\mathrm{THQ}}_{i}$$

#### 3.6.3. Carcinogenic Risk (CR) and Cumulative Cancer Risk (CCR)

The formula below was used to calculate CR over a lifetime exposure to contaminated sweets containing Al, Ni, Cr, As, Cu, Cd, and Pb. Each metal carries a distinctive cancer slope factor (CSF); for instance, Al = 0.0014, Cr = 0.5, Ni = 0.91, As = 1.5, Cd = 0.38, and Pb = 0.0085 mg/kg/day, indicating their varying carcinogenic potential. A value of less than 10^−3^ represents a serious cancer threat and necessitates higher priority care and 10^−4^ is a threshold [59].

$$\mathrm{CR}=\frac{\mathrm{EF}\;\times \mathrm{ED}\;\times \mathrm{IR}\;\times \mathrm{MC}\times \mathrm{CSF}}{\mathrm{BW}\;\times {\mathrm{AT}}_{\mathrm{cancer}}}\times 10^{-3}$$

The AT_cancer_ is averaging time for carcinogens (365 days/year × 66.4 years, is average life time of Pakistan). Whereas CCR can be calculated by following formula

$$\mathrm{CCR}=\overset{n}{\underset{i=1}{\sum }}{\mathrm{CR}}_{i}\;$$

## 4. Conclusions

The data from this study were used to assess the EMs and PTMs in 100 wheat-based sweet samples. The results of the current investigation have revealed discrepancies in the metal composition of wheat-based sweets. The concentration trend of EMs and PTMs was Ca > Mg > Fe > Mn > Zn > Co > Cu > Cr > Ni > Al > Pb > Cd. The mean concentration of Ni was in different sweet samples was obtained in the order SL > SPe > ST > SG > SRP > SRW = SP > SDB > SS > SB. Ni is used as catalyst in hydrogenation of oil into ghee and trace amount of Ni in ghee can be contaminated wheat-based sweets because these sweets are cooked in ghee. The PCC highlighted the significant positive correlation between the metals like Ca-Mg (r = 0.76), Cu-Ca (r = 0.73), Zn-Fe (r = 0.64), Cr-Mn (r = 0.48), Cr-Al (r = 0.43), Ni-Zn (r = 0.38), Ni-Co (r = 0.38), Cd-Fe (r = 0.62), and Pb-Cu (r = 0.38). Significant correlations were found between metals within the same clusters and principal components indicating that the results of PCA, HCA, and PCC were all supportive. The highest values of THQ was obtained for Al in SP and SL samples respectively. The HI values were more than the threshold value 1 in SP samples. The findings showed that the customers would be exposed to major potential health risks over the course of their entire lives if they consume “Patisa” sweets. The CR value found for Ni at the threshold level of cancer risk for all samples except SB and SS samples. Therefore, the evaluated sweets may be believed unsafe for consumers if frequently ingested. This research will greatly enhance our understanding of how EMs and PTMs are transferred and moved across various matrices. By gaining a clearer insight into these processes, we can better grasp the pathways via which humans are exposed to these metals. Globally, controlling food contamination has become a significant issue, given the potential risks it poses to human health. It is imperative to implement a system of consistent monitoring for contaminants in food products, which is particularly crucial for developing countries. Hence, it is of utmost importance for the responsible authorities to diligently oversee and track the sources of the essential and potentially toxic elements found in wheat-based sweets, as they could potentially jeopardize our well-being.

## Figures and Tables

**Table 1:** Measured and certified values of the metals in SRM 2384.

Metal	Certified Value (mg/kg)	$\mathrm{Measured}\;\mathrm{Value}\;(\bar{x}\pm \;ts/\surd n$)	% Recovery
Mg	2610 ± 120	2600 ± 100	99.6
Ca	840 ± 74	850 ± 70	101.2
Mn	20.8 ± 1.3	21.1 ± 1.5	101.4
Cu	23.9 ± 1.0	23.7 ± 0.3	99.2
Zn	37.6 ± 1.9	37.9 ± 0.8	100.8
Fe	132 ± 11	135 ± 20	102.3
Cd	0.0734 ± 0.0077	0.072 ± 0.004	98.1
Pb	0.0357 ± 0.0046	0.036 ± 0.005	100.8

($\bar{x}\pm \;ts/\surd n$) = mean ± CI (*p* < 0.05, *n* = 6), CI = Confidence interval.

**Table 2:** EMs and PTMs in wheat-based sweet samples (mg/kg, *n* = 3, mean ± SD ^a^).

Sample	SG	SB	SRW	SRP	SS	SP	SL	SDB	ST	SPe
Minimum	9.9 ± 0.1	43.2 ± 0.2	2.6 ± 0.1	6.3 ± 0.1	2.9 ± 0.1	16.4 ± 0.3	12.6 ± 0.6	13.7 ± 0.1	15.4 ± 0.2	27.8 ± 0.4
Maximum	46.3 ± 0.7	85.3 ± 0.4	22.8 ± 0.4	29.4 ± 0.9	20.6 ± 0.8	29.6 ± 0.5	38.6 ± 0.4	27.7 ± 0.4	27.8 ± 0.6	73.8 ± 0.9
Mean (Mg, *n* = 10)	26.8 ± 0.6	65.7 ± 0.1	13.6 ± 0.8	14.8 ± 0.4	12.7 ± 0.2	22.2 ± 0.6	23.6 ± 0.6	22.0 ± 0.7	21.5 ± 0.7	42.8 ± 0.3
Minimum	10.6 ± 0.1	28.5 ± 0.8	82.2 ± 0.4	10.6 ± 0.4	37.9 ± 0.8	42.9 ± 0.7	11.1 ± 0.2	21.8 ± 0.4	16.8 ± 0.8	16.0 ± 0.5
Maximum	353.0 ± 1.0	630.0 ± 1.0	160.0 ± 2.0	79.9 ± 0.9	68.6 ± 0.6	66.8 ± 0.7	39.8 ± 0.6	44.1 ± 0.3	43.7 ± 0.4	337.0 ± 1.0
Mean (Ca, *n* = 10)	93.0 ± 1.0	209.1 ± 0.9	94.2 ± 0.7	41.9 ± 0.4	56.2 ± 0.4	54.0 ± 0.5	24.0 ± 0.8	30.2 ± 0.4	34.4 ± 0.6	60.4 ± 0.5
Minimum	3.7 ± 0.1	5.7 ± 0.1	0.55 ± 0.03	0.11 ± 0.01	5.3 ± 0.2	3.7 ± 0.1	2.1 ± 0.1	0.41 ± 0.02	0.46 ± 0.02	1.5 ± 0.2
Maximum	9.4 ± 0.1	8.6 ± 0.3	4.1 ± 0.1	4.4 ± 0.2	10.8 ± 0.6	14.5 ± 0.4	8.1 ± 0.2	5.6 ± 0.2	7.3 ± 0.2	5.8 ± 0.2
Mean (Mn, *n* = 10)	7.1 ± 0.1	6.8 ± 0.3	1.8 ± 0.1	1.3 ± 0.1	8.4 ± 0.1	9.6 ± 0.1	5.0 ± 0.1	3.3 ± 0.1	4.2 ± 0.2	3.6 ± 0.2
Minimum	9.6 ± 0.1	0.12 ± 0.02	3.6 ± 0.2	0.20 ± 0.02	2.5 ± 0.2	1.2 ± 0.02	0.84 ± 0.08	32.6 ± 0.9	3.2 ± 0.1	19.6 ± 0.5
Maximum	23.2 ± 0.2	10.9 ± 0.4	19.2 ± 0.8	48.8 ± 0.4	164.0 ± 1.0	100.5 ± 0.9	167.0 ± 1.0	239.6 ± 1.0	336.6 ± 2.0	97.4 ± 1.0
Mean (Fe, *n* = 10)	17.1 ± 0.7	4.6 ± 0.6	9.1 ± 0.3	24.0 ± 0.7	39.6 ± 0.2	46.0 ± 1.0	48.0 ± 1.0	111.2 ± 2.0	77.0 ± 1.0	67.0 ± 1.0
Minimum	6.5 ± 0.1	6.4 ± 0.2	2.3 ±0.1	2.4 ± 0.1	2.7 ± 0.1	0.25 ± 0.02	1.1 ± 0.1	1.4 ± 0.2	1.1 ± 0.1	1.3 ± 0.1
Maximum	10.5 ± 1.0	12.0 ± 1.0	4.6 ± 0.3	28.6 ± 0.3	5.0 ± 0.2	6.2 ± 0.2	5.6 ± 0.3	4.1 ± 0.2	3.5 ± 0.2	3.4 ± 0.1
Mean (Cu, *n* = 10)	8.1 ± 0.2	8.6 ± 0.6	3.7 ± 0.2	5.8 ± 0.2	3.8 ± 0.1	4.2 ± 0.1	4.1 ± 0.1	2.4 ± 0.2	2.1 ± 0.1	2.6 ± 0.1
Minimum	1.8 ± 0.1	3.0 ± 0.3	5.5 ± 0.3	6.6 ± 0.2	4.8 ± 0.6	4.8 ± 0.2	8.2 ± 0.5	4.3 ± 0.2	4.0 ± 0.1	7.0 ± 0.3
Maximum	4.6 ± 0.3	12.7 ± 0.7	10.7 ± 0.5	13.2 ± 0.2	16.1 ± 0.5	15.5 ± 0.8	12.8 ± 0.6	17.4 ± 0.9	67.7 ± 0.7	22.4 ± 0.9
Mean (Zn, *n* = 10)	2.6 ± 0.1	5.7 ± 0.8	7.6 ± 0.5	9.2 ± 0.9	11.5 ± 0.7	9.1 ± 0.4	10.3 ± 0.8	10.0 ± 1.0	19.0 ± 1.0	15.7 ± 1.0
Minimum	3.2 ± 0.4	1.2 ± 0.1	0.12 ± 0.03	0.11 ± 0.03	2.6 ± 0.1	0.18 ± 0.05	5.0 ± 0.1	0.12 ± 0.05	0.16 ± 0.05	0.17 ± 0.05
Maximum	4.3 ± 0.2	2.1 ± 0.1	2.6 ± 0.1	1.4 ± 0.1	8.2 ± 0.5	0.87 ± 0.04	14.3 ± 1.0	0.57 ± 0.04	0.98 ± 0.04	0.65 ± 0.05
Mean (Co, *n* = 10)	3.9 ± 0.2	1.6 ± 0.4	1.5 ± 0.4	0.78 ± 0.03	6.5 ± 0.4	0.41 ± 0.03	9.0 ± 1.0	0.32 ± 0.03	0.45 ± 0.03	0.43 ± 0.04
Minimum	0.11 ± 0.03	0.18 ± 0.02	0.18 ± 0.05	0.25 ± 0.05	0.21 ± 0.02	0.12 ± 0.01	0.58 ± 0.02	0.11 ± 0.04	0.47 ± 0.07	0.33 ± 0.02
Maximum	0.95 ± 0.05	0.98 ± 0.04	0.87 ± 0.06	1.0 ± 0.1	0.65 ± 0.05	1.1 ± 0.1	1.1 ± 0.03	0.57 ± 0.06	0.95 ± 0.05	0.87 ± 0.05
Mean (Al, *n* = 10)	0.41 ± 0.06	0.51 ± 0.04	0.38 ± 0.03	0.62 ± 0.04	0.35 ± 0.05	0.87 ± 0.03	0.86 ± 0.03	0.32 ± 0.03	0.69 ± 0.04	0.46 ± 0.03
Minimum	0.14 ± 0.02	0.16 ± 0.03	0.25 ± 0.05	0.14 ± 0.05	1.1 ± 0.1	3.2 ± 0.1	0.16 ± 0.05	1.1 ± 0.1	1.5 ± 0.2	0.11 ± 0.01
Maximum	0.87 ± 0.06	0.54 ± 0.03	2.4 ± 0.2	1.1 ± 0.2	2.3 ± 0.1	7.5 ± 0.4	0.25 ± 0.05	1.5 ± 0.1	3.1 ± 0.1	1.0 ± 0.04
Mean (Cr, *n* = 10)	0.39 ± 0.04	0.23 ± 0.03	1.5 ± 0.1	0.64 ± 0.03	1.6 ± 0.1	5.7 ± 0.7	0.17 ± 0.04	1.3 ± 0.1	2.2 ± 0.1	0.47 ± 0.03
Minimum	0.13 ± 0.02	0.14 ± 0.01	0.12 ± 0.01	0.14 ± 0.02	0.15 ± 0.02	0.11 ± 0.03	1.2 ± 0.1	0.32 ± 0.03	0.14 ± 0.02	1.0 ± 0.1
Maximum	1.3 ± 0.1	0.87 ± 0.02	1.1 ± 0.1	0.92 ± 0.06	0.67 ± 0.04	0.85 ± 0.05	2.1 ± 0.1	0.61 ± 0.03	1.5 ± 0.1	1.6 ± 0.1
Mean (Ni, *n* = 10)	0.64 ± 0.03	0.27 ± 0.03	0.49 ± 0.03	0.56 ± 0.04	0.36 ± 0.03	0.49 ± 0.04	1.5 ± 0.1	0.45 ± 0.03	0.72 ± 0.04	1.4 ± 0.1
Minimum	ND	ND	ND	ND	ND	ND	ND	ND	ND	ND
Maximum	ND	ND	ND	ND	ND	ND	ND	ND	ND	ND
Mean (As, *n* = 10)	ND	ND	ND	ND	ND	ND	ND	ND	ND	ND
Minimum	0.11 ± 0.01	0.13 ± 0.01	0.13 ± 0.01	0.11 ± 0.01	0.11 ± 0.01	0.12 ± 0.01	0.13 ± 0.01	0.14 ± 0.02	0.15 ± 0.02	0.16 ± 0.02
Maximum	0.31 ± 0.04	0.98 ± 0.04	0.47 ± 0.03	0.54 ± 0.05	0.44 ± 0.06	0.65 ± 0.15	1.2 ± 0.1	1.0 ± 0.1	0.68 ± 0.04	0.48 ± 0.04
Mean (Cd, *n* = 10)	0.16 ± 0.03	0.35 ± 0.03	0.24 ± 0.02	0.28 ± 0.03	0.17 ± 0.03	0.23 ± 0.03	0.46 ± 0.03	0.56 ± 0.03	0.31 ± 0.03	0.31 ± 0.03
Minimum	0.18 ± 0.04	0.17 ± 0.03	0.25 ± 0.05	0.24 ± 0.04	0.16 ± 0.04	0.21 ± 0.04	0.15 ± 0.01	0.28 ± 0.04	0.24 ± 0.06	0.14 ± 0.03
Maximum	0.87 ± 0.04	2.1 ± 0.2	0.64 ± 0.04	1.2 ± 0.1	0.50 ± 0.1	0.72 ± 0.06	0.24 ± 0.07	1.0 ± 0.2	0.90 ± 0.1	0.87 ± 0.06
Mean (Pb, *n* = 10)	0.38 ± 0.02	0.90 ± 0.1	0.48 ± 0.04	0.72 ± 0.03	0.18 ± 0.03	0.56 ± 0.04	0.14 ± 0.03	0.58 ± 0.05	0.60 ± 0.04	0.32 ± 0.08

^a^ Each sample was analysed in triplicate, *n* = 10, mean of ten samples, ND = not detected.

**Table 3:** Results of EDI of EMs and PTMs in wheat-based sweets.

Metals	SG	SB	SRW	SRP	SS	SP	SL	SDB	ST	SPe
Mg	9.57 × 10^−3^	2.35 × 10^−2^	4.84 × 10^−3^	5.30 × 10^−3^	4.54 × 10^−3^	7.92 × 10^−3^	8.42 × 10^−3^	7.85 × 10^−3^	7.68 × 10^−3^	1.53 × 10^−2^
Ca	3.31 × 10^−2^	7.47 × 10^−2^	3.36 × 10^−2^	1.49 × 10^−2^	2.01 × 10^−2^	1.93 × 10^−2^	8.58 × 10^−3^	1.08 × 10^−2^	1.23 × 10^−2^	2.16 × 10^−2^
Mn	2.52 × 10^−3^	2.42 × 10^−3^	6.54 × 10^−4^	4.71 × 10^−4^	3.01 × 10^−3^	3.43 × 10^−3^	1.77 × 10^−3^	1.17 × 10^−3^	1.49 × 10^−3^	1.27 × 10^−3^
Fe	6.10 × 10^−3^	1.62 × 10^−3^	3.25 × 10^−3^	8.59 × 10^−3^	1.41 × 10^−2^	1.63 × 10^−2^	1.73 × 10^−2^	3.97 × 10^−2^	2.75 × 10^−2^	2.39 × 10^−2^
Cu	2.88 × 10^−3^	3.07 × 10^−3^	1.32 × 10^−3^	2.07 × 10^−3^	1.34 × 10^−3^	1.49 × 10^−3^	1.48 × 10^−3^	8.68 × 10^−4^	7.57 × 10^−4^	9.36 × 10^−4^
Zn	9.29 × 10^−4^	2.02 × 10^−3^	2.72 × 10^−3^	3.27 × 10^−3^	4.10 × 10^−3^	3.25 × 10^−3^	3.68 × 10^−3^	3.65 × 10^−3^	6.91 × 10^−3^	5.60 × 10^−3^
Co	1.41 × 10^−3^	5.61 × 10^−4^	5.39 × 10^−4^	2.79 × 10^−4^	2.31 × 10^−3^	1.46 × 10^−4^	3.19 × 10^−3^	1.14 × 10^−4^	1.61 × 10^−4^	1.54 × 10^−4^
Al	1.46 × 10^−4^	1.82 × 10^−4^	1.36 × 10^−4^	2.21 × 10^−4^	1.27 × 10^−4^	3.11 × 10^−4^	3.07 × 10^−4^	1.14 × 10^−4^	2.46 × 10^−4^	1.64 × 10^−4^
Cr	1.39 × 10^−4^	8.21 × 10^−5^	5.25 × 10^−4^	2.29 × 10^−4^	5.79 × 10^−4^	2.05 × 10^−3^	6.07 × 10^−5^	4.54 × 10^−4^	7.82 × 10^−4^	1.68 × 10^−4^
Ni	2.29 × 10^−4^	9.64 × 10^−5^	1.75 × 10^−4^	2.00 × 10^−4^	1.31 × 10^−4^	1.75 × 10^−4^	5.50 × 10^−4^	1.61 × 10^−4^	2.57 × 10^−4^	4.89 × 10^−4^
Cd	5.71 × 10^−5^	1.25 × 10^−4^	8.57 × 10^−5^	1.00 × 10^−4^	5.96 × 10^−5^	8.21 × 10^−5^	1.64 × 10^−4^	2.00 × 10^−4^	1.11 × 10^−4^	1.11 × 10^−4^
Pb	1.36 × 10^−4^	3.29 × 10^−4^	1.71 × 10^−4^	2.57 × 10^−4^	6.57 × 10^−5^	2.00 × 10^−4^	5.00 × 10^−5^	2.07 × 10^−4^	2.14 × 10^−4^	1.14 × 10^−4^

## Data Availability

This study did not report any data.

## Supplementary Materials

The following supporting information can be downloaded at: https://www.mdpi.com/article/10.3390/molecules28217365/s1, Table S1: Parameters of regression equation, detection limits and precision studies; Table S2: PCA results of EMs and PTMs in sweets; Table S3: Eigen values of correlation matrix of EMs and PTMs in sweets; Table S4: Results of THQ of EMs and PTMs in sweets; Table S5: Results of CR of PTMs in sweets; Table S6: Results of HI and CCR of PTMs in sweets.
