# Supplementary material for: Exposure Assessment of Essential and Potentially Toxic Metals in Wheat-Based Sweets for Human Consumption: Multivariate Analysis and Risk Evaluation Studies
Source: Molecules. 2023 Oct 31;28(21):7365. doi: 10.3390/molecules28217365 (PMC10650165; doi:10.3390/molecules28217365)
Supplement: Supplementary file 1 [file molecules-28-07365-s001.zip › molecules-2668986-supplementary.pdf]

Supplementary Data

Exposure Assessment of Essential and Potentially Toxic Metals in Wheat-Based Sweets for Human Consumption: Multivariate Analysis and Risk Evaluation Studies

Mahmood Ahmed [1,*], Syed Salman Shafqat [1], Amna Javed [1], Mudassar Sanaullah [1], Abdul Shakoor [2], Muhammad Imtiaz Shafiq [3,4], Syeda Kiran Shahzadi [5], Tanveer A. Wani [6] and Seema Zargar [7]

[1] Department of Chemistry, Division of Science and Technology, University of Education, College Road, Lahore 54770, Pakistan; salman.shafqat@ue.edu.pk (S.S.S.); amnajaved0107@gmail.com (A.J.); mudassarsanaullah312@gmail.com (M.S.)
[2] CSH Pharmaceuticals (Pvt.) Ltd., Ferozepur Road, Lahore 54000, Pakistan; chemistpbu@gmail.com
[3] School of Chemistry, University of the Punjab, Lahore 54590, Pakistan; imtiaz.ibb@pu.edu.pk
[4] Center for Bioinformatics and Drug Designing, University of the Punjab, Lahore 54590, Pakistan
[5] Department of Physiology, Faculty of Medicine and Health Sciences, McGill University, Montreal, QC H3A 0G4, Canada; kiran.syyed@gmail.com
[6] Department of Pharmaceutical Chemistry, College of Pharmacy, King Saud University, P.O. Box 2457, Riyadh 11451, Saudi Arabia; twani@ksu.edu.sa
[7] Department of Biochemistry, College of Science, King Saud University, P.O. Box 222452, Riyadh 11451, Saudi Arabia; szargar@ksu.edu.sa
* Correspondence: mahmoodresearchscholar@gmail.com or mahmood.ahmed@ue.edu.pk

Supplementary Table S1. Parameters of regression equation, detection limits and precision studies

Element	Coefficient of Determination (r^2)	Intercept (c)	Slope (m)	LOD (µg/g)	LOQ (µg /g)
Mn	0.9930	0.0048	0.0364	0.27	0.91
Fe	0.9922	0.0022	0.0133	0.23	0.77
Cu	0.9984	0.0038	0.0694	0.11	0.36
Zn	0.9906	0.0056	0.296	0.14	0.47
Mg	0.9902	0.0084	0.31	0.20	0.67
Ca	0.9824	0.0079	0.0059	0.34	0.14
Co	0.9992	40.541	78.649	0.0001	0.0003
Cr	0.9999	8.1081	150.27	0.001	0.003
Cd	0.9901	6908.1	1560.3	0.0004	0.0013
Pb	0.9977	108.11	70.27	0.008	0.027
Al	0.9992	86.486	96.216	0.01	0.033
Ni	0.9979	437.84	229.59	0.001	0.003

Table S2. PCA results of EMs and PTMs in sweets

Metals	PC1	PC2
Loading values	33.78%	19.05%
Mg	0.27096	0.18764
Ca	0.45129	0.10081
Mn	0.14437	-0.30847
Fe	-0.3883	0.2579
Cu	0.45031	-0.13989
Zn	-0.37381	0.18695
Co	-0.0442	-0.57377
Al	-0.12007	-0.1509
Cr	-0.08553	0.03721
Ni	-0.27954	-0.22713
Cd	-0.19103	0.28924
Pb	0.26546	0.49982

Table S3. Eigen values of correlation matrix of EMs and PTMs in sweets

Eigenvalue	Percentage of Variance	Cumulative
4.05321	33.78%	33.78%
2.2861	19.05%	52.83%
2.03818	16.98%	69.81%
1.44212	12.02%	81.83%
0.89343	7.45%	89.28%
0.75909	6.33%	95.60%
0.3124	2.60%	98.20%
0.20839	1.74%	99.94%
0.0071	0.06%	100.00%
0	0.00%	100.00%

Table S4. Results of THQ of EMs and PTMs in sweets

Metals	SG	SB	SRW	SRP	SS	SP	SL	SDB	ST	SPe
Mn	1.28E-02	1.23E-02	3.33E-03	2.40E-03	1.53E-02	1.75E-02	9.01E-03	5.95E-03	7.58E-03	6.46E-03
Fe	6.21E-03	1.65E-03	3.31E-03	8.74E-03	1.43E-02	1.66E-02	1.76E-02	4.04E-02	2.80E-02	2.43E-02
Cu	5.13E-02	5.47E-02	2.35E-02	3.69E-02	2.39E-02	2.65E-02	2.64E-02	1.55E-02	1.35E-02	1.67E-02
Zn	2.21E-03	4.80E-03	6.46E-03	7.76E-03	9.74E-03	7.72E-03	8.74E-03	8.67E-03	1.64E-02	1.33E-02
Co	5.02E-02	2.00E-02	1.92E-02	9.94E-03	8.23E-02	5.20E-03	1.14E-01	4.06E-03	5.73E-03	5.48E-03
Al	2.60E-01	3.24E-01	2.42E-01	3.94E-01	2.26E-01	5.54E-01	5.47E-01	2.03E-01	4.38E-01	2.92E-01
Cr	3.30E-02	1.95E-02	1.25E-01	5.44E-02	1.37E-01	4.87E-01	1.44E-02	1.08E-01	1.86E-01	3.99E-02
Ni	8.16E-03	3.43E-03	6.23E-03	7.12E-03	4.67E-03	6.23E-03	1.96E-02	5.73E-03	9.15E-03	1.74E-02
Cd	4.07E-02	8.90E-02	6.10E-02	7.12E-02	4.25E-02	5.85E-02	1.17E-01	1.42E-01	7.91E-02	7.91E-02
Pb	2.77E-02	6.70E-02	3.48E-02	5.23E-02	1.34E-02	4.07E-02	1.02E-02	4.21E-02	4.36E-02	2.32E-02

Table S5. Results of CR of PTMs in sweets

Metals	Al	Cr	Ni	Cd	Pb
SG	1.46E-07	4.96E-05	1.48E-04	1.55E-05	8.22E-07
SB	1.82E-07	2.93E-05	6.25E-05	3.38E-05	1.99E-06
SRW	1.35E-07	1.87E-04	1.13E-04	2.32E-05	1.04E-06
SRP	2.21E-07	8.14E-05	1.30E-04	2.71E-05	1.56E-06
SS	1.27E-07	2.06E-04	8.50E-05	1.61E-05	3.98E-07
SP	3.10E-07	7.30E-04	1.13E-04	2.22E-05	1.21E-06
SL	3.06E-07	2.16E-05	3.57E-04	4.45E-05	3.03E-07
SDB	1.14E-07	1.62E-04	1.04E-04	5.41E-05	1.25E-06
ST	2.46E-07	2.79E-04	1.67E-04	3.00E-05	1.30E-06
SPe	1.64E-07	5.98E-05	3.17E-04	3.00E-05	6.92E-07

Table S6. Results of HI and CCR of PTMs in sweets

Sample	HI		CCR	
SG	0.492	No significant hazard	2.14E-04	No carcinogenic risk
SB	0.596	No significant hazard	1.28E-04	No carcinogenic risk
SRW	0.525	No significant hazard	3.24E-04	No carcinogenic risk
SRP	0.645	No significant hazard	2.40E-04	No carcinogenic risk
SS	0.569	No significant hazard	3.08E-04	No carcinogenic risk
SP	1.22	Significant hazard	8.67E-04	No carcinogenic risk
SL	0.884	No significant hazard	4.24E-04	No carcinogenic risk
SDB	0.575	No significant hazard	3.21E-04	No carcinogenic risk
ST	0.827	No significant hazard	4.78E-04	No carcinogenic risk
SPe	0.518	No significant hazard	4.08E-04	No carcinogenic risk
